# *N*,*N*′-Dimethyl-*N*,*N*′-bis­(pyridin-2-yl)methane­diamine

**DOI:** 10.1107/S1600536811046320

**Published:** 2011-11-09

**Authors:** Moon-Sun Lee, Minyoung Yoon, O-bong Yang, Dong-Heon Lee, Gyungse Park

**Affiliations:** a Department of Chemistry, Chonbuk National University, Jeonju, Chonbuk 561-756, Republic of Korea; b National Creative Research Initiative Center for Smart Supramolecules (CSS), Department of Chemistry and Division of Advanced Materials Science, Pohang University of Science and Technology (POSTECH), Pohang 790-784, Republic of Korea; c School of Semiconductor and Chemical Engineering & Solar Energy Research Center, Chonbuk National University, Jeonju, Chonbuk 561-756, Republic of Korea; d The Department of Chemistry, College of Science and Technology, Kunsan National University, 68 Miryong-Dong, Kusan, Jeollabuk-Do 573-701, Republic of Korea

## Abstract

The title compound, C_13_H_16_N_4_, consists of two pyridine rings which are linked by an *N*,*N*′-dimethyl­methane­amine chain. The pyridine rings adopt a twist conformation and the dihedral angle between them is 60.85 (5)°. The crystal packing is stabilized by weak C—H⋯π inter­actions.

## Related literature

For the synthesis of the title compound, see: Kahn *et al.* (1945 ▶). For applications of heteroaromatic amines, see: Mehrkhodavandi & Schrock (2001 ▶); Hall *et al.* (1998 ▶); Lee (2003 ▶).
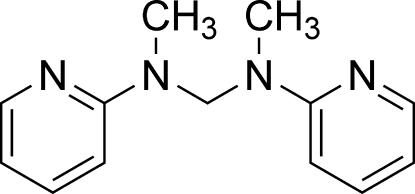


## Experimental

### 

#### Crystal data

C_13_H_16_N_4_
*M* *_r_* = 228.30
Monoclinic, 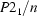

*a* = 11.6652 (7) Å
*b* = 8.3921 (5) Å
*c* = 12.8966 (7) Å
β = 106.634 (2)°
*V* = 1209.69 (12) Å^3^
*Z* = 4
Mo *K*α radiation
μ = 0.08 mm^−1^
*T* = 100 K
0.12 × 0.08 × 0.08 mm

#### Data collection

Bruker APEXII diffractometer
Absorption correction: multi-scan (*SADABS*; Sheldrick, 1996 ▶) *T*_min_ = 0.963, *T*_max_ = 0.986
27844 measured reflections
2516 independent reflections
2105 reflections with *I* > 2σ(*I*)
*R*_int_ = 0.050

#### Refinement

*R*[*F* ^2^ > 2σ(*F* ^2^)] = 0.038
*wR*(*F* ^2^) = 0.125
*S* = 0.96
2516 reflections
218 parameters
All H-atom parameters refined
Δρ_max_ = 0.23 e Å^−3^
Δρ_min_ = −0.21 e Å^−3^

### 

Data collection: *APEX2* (Bruker, 2007 ▶); cell refinement: *SAINT* (Bruker, 2007 ▶); data reduction: *SAINT*; program(s) used to solve structure: *SHELXS97* (Sheldrick, 2008 ▶); program(s) used to refine structure: *SHELXL97* (Sheldrick, 2008 ▶); molecular graphics: *DIAMOND* (Brandenburg & Putz, 2005 ▶); software used to prepare material for publication: *SHELXL97*.

## Supplementary Material

Crystal structure: contains datablock(s) I, global. DOI: 10.1107/S1600536811046320/bx2378sup1.cif

Structure factors: contains datablock(s) I. DOI: 10.1107/S1600536811046320/bx2378Isup2.hkl

Supplementary material file. DOI: 10.1107/S1600536811046320/bx2378Isup3.cml

Additional supplementary materials:  crystallographic information; 3D view; checkCIF report

## Figures and Tables

**Table 1:** Hydrogen-bond geometry (Å, °) *Cg*1 and *Cg*2 are the centroids of the N1/C1–C5 and N4/C9–C15 rings, respectively.

*D*—H⋯*A*	*D*—H	H⋯*A*	*D*⋯*A*	*D*—H⋯*A*
C1—H1⋯*Cg*2^i^	0.99 (2)	2.64 (2)	3.484 (1)	143 (1)
C6—H7⋯*Cg*2^ii^	1.00 (2)	2.75 (2)	3.545 (2)	137 (1)
C10—H13⋯*Cg*1^iii^	0.95 (2)	2.90 (1)	3.637 (1)	135 (1)

Symmetry codes: (i) 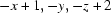; (ii) 
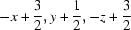
; (iii) 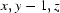.

**Fig. 1.**
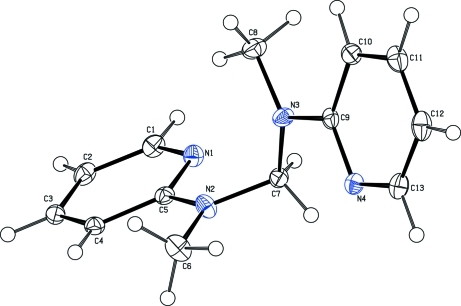
A view of the title compound showing the labelling of the atoms. Displacement ellipsoids are shown at the 50% probability level.

**Fig. 2.**
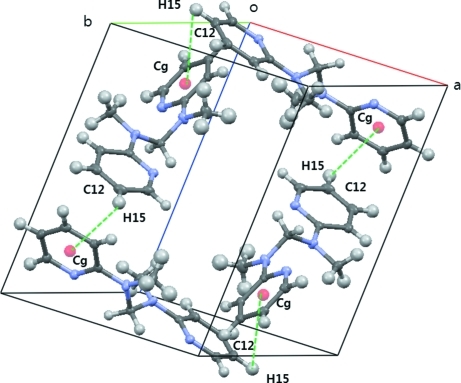
Part of the crystal structure of (I) showing the formation of a C—H···π interactions.

## supplementary crystallographic information

### Comment

Heteroaromatic amines based metal complexes have been extensively studied due to their numerous potential applications as catalysts, drugs, biomimetic chemistry, and so on. (Mehrkhodavandi, *et al.*, (2001) , Hall, *et al.*,(1998), Lee, (2003). We are interested in the use of chelates containing pyridylamine. We report here the crystal structure of the title compound, Fig. 1, which consists of two 2-pyridyl rings which are linked together by a *N*,*N'*-dimethylmethaneamine chain .The pyridine rings adopt a twist conformation and the dihedral angle between them is 60.85 (5)°. The crystal packing is stabilized by weak C—H···π interactions, Fig. 2, Table 1.

### Experimental

*N*,*N*'-dimethyl-*N*,*N*'-di(pyridin-2-yl)methanediamine was prepared by a reported method, Kahn, *et al.*, (1945). A solution of 2-(methylamino)pyridine (5.00 g, 4.62 × 10 ^-2^ mol) in water (50 ml) was added dropwise to 37% formaldehyde solution (1.83 ml, 2.31 × 10^-2 ^mol) at 0°C. The reation mixture was stirred at room temperature for overnight and the white precipitate formed was filtered,washed with water and dried. It was dissolved in acetone, dried over MgSO_4_ and concentrated. It was recrystallized in acetonitrile. Yield: 92% (4.88 g)

### Refinement

All H atoms were geometrically positioned and refined using a riding model, with C—H = 0.95 Å for the aryl, 0.99 Å for the methylene, and 0.00 Å for the methyl H atoms, respectively, and with *U*_iso_(H) = 1.2Ueq(C) for the aryl and methylene H atoms, and 1.5Ueq(C) for the methyl H atoms.

### Crystal data

**Table:**

C_13_H_16_N_4_	*F*(000) = 488
*M**_r_* = 228.30	*D*_x_ = 1.254 Mg m^−^^3^
Monoclinic, *P*2_1_/*n*	Mo *K*α radiation, λ = 0.71073 Å
Hall symbol: -P2yn	Cell parameters from 2516 reflections
*a* = 11.6652 (7) Å	θ = 2.1–26.5°
*b* = 8.3921 (5) Å	µ = 0.08 mm^−^^1^
*c* = 12.8966 (7) Å	*T* = 100 K
β = 106.634 (2)°	Rod, colourless
*V* = 1209.69 (12) Å^3^	0.12 × 0.08 × 0.08 mm
*Z* = 4	

### Data collection

**Table:**

Bruker APEXII diffractometer	2105 reflections with *I* > 2σ(*I*)
Radiation source: fine-focus sealed tube	*R*_int_ = 0.050
graphite	θ_max_ = 26.5°, θ_min_ = 2.1°
θ/2φ scans	*h* = −14→14
Absorption correction: multi-scan (*SADABS*; Sheldrick, 1996)	*k* = −10→10
*T*_min_ = 0.963, *T*_max_ = 0.986	*l* = −16→14
27844 measured reflections	4 standard reflections every 30 min
2516 independent reflections	intensity decay: 0.0%

### Refinement

**Table:**

Refinement on *F*^2^	Primary atom site location: structure-invariant direct methods
Least-squares matrix: full	Secondary atom site location: difference Fourier map
*R*[*F*^2^ > 2σ(*F*^2^)] = 0.038	Hydrogen site location: inferred from neighbouring sites
*wR*(*F*^2^) = 0.125	All H-atom parameters refined
*S* = 0.96	*w* = 1/[σ^2^(*F*_o_^2^) + (0.1*P*)^2^] where *P* = (*F*_o_^2^ + 2*F*_c_^2^)/3
2516 reflections	(Δ/σ)_max_ < 0.001
218 parameters	Δρ_max_ = 0.23 e Å^−^^3^
0 restraints	Δρ_min_ = −0.21 e Å^−^^3^

### Special details

**Table:**

Geometry. All e.s.d.'s (except the e.s.d. in the dihedral angle between two l.s. planes) are estimated using the full covariance matrix. The cell e.s.d.'s are taken into account individually in the estimation of e.s.d.'s in distances, angles and torsion angles; correlations between e.s.d.'s in cell parameters are only used when they are defined by crystal symmetry. An approximate (isotropic) treatment of cell e.s.d.'s is used for estimating e.s.d.'s involving l.s. planes.
Refinement. Refinement of *F*^2^ against ALL reflections. The weighted *R*-factor *wR* and goodness of fit *S* are based on *F*^2^, conventional *R*-factors *R* are based on *F*, with *F* set to zero for negative *F*^2^. The threshold expression of *F*^2^ > σ(*F*^2^) is used only for calculating *R*-factors(gt) *etc*. and is not relevant to the choice of reflections for refinement. *R*-factors based on *F*^2^ are statistically about twice as large as those based on *F*, and *R*- factors based on ALL data will be even larger.

### Fractional atomic coordinates and isotropic or equivalent isotropic displacement parameters (Å^2^)

**Table:**

	*x*	*y*	*z*	*U*_iso_*/*U*_eq_	
N1	0.43474 (8)	0.22148 (11)	0.82931 (7)	0.0194 (3)	
N2	0.61745 (8)	0.11262 (11)	0.82658 (8)	0.0203 (3)	
N3	0.59838 (8)	−0.15394 (11)	0.89105 (8)	0.0189 (3)	
N4	0.79220 (8)	−0.22454 (12)	0.98754 (7)	0.0182 (3)	
C1	0.34526 (10)	0.32394 (15)	0.78856 (9)	0.0211 (3)	
C2	0.33876 (11)	0.42500 (14)	0.70242 (9)	0.0214 (3)	
C3	0.43186 (10)	0.41792 (14)	0.65514 (9)	0.0200 (3)	
C4	0.52555 (10)	0.31431 (14)	0.69472 (9)	0.0173 (3)	
C5	0.52473 (10)	0.21664 (13)	0.78366 (9)	0.0160 (3)	
C6	0.71691 (12)	0.09942 (17)	0.78056 (12)	0.0272 (3)	
C7	0.62117 (10)	0.01339 (13)	0.91939 (9)	0.0173 (3)	
C8	0.47475 (11)	−0.19594 (16)	0.83643 (11)	0.0240 (3)	
C9	0.67862 (10)	−0.27049 (13)	0.94043 (8)	0.0163 (3)	
C10	0.64315 (11)	−0.43154 (14)	0.93928 (9)	0.0205 (3)	
C11	0.72811 (12)	−0.54307 (15)	0.98755 (9)	0.0248 (3)	
C12	0.84533 (12)	−0.49686 (15)	1.03581 (9)	0.0255 (3)	
C13	0.87199 (11)	−0.33721 (14)	1.03368 (9)	0.0221 (3)	
H1	0.2816 (13)	0.3259 (16)	0.8256 (12)	0.031 (4)*	
H2	0.2701 (12)	0.4955 (17)	0.6790 (10)	0.029 (4)*	
H3	0.4313 (12)	0.4841 (17)	0.5946 (11)	0.028 (4)*	
H4	0.5883 (13)	0.3065 (16)	0.6629 (11)	0.025 (3)*	
H5	0.7601 (15)	0.201 (2)	0.7856 (14)	0.048 (5)*	
H6	0.7696 (15)	0.022 (2)	0.8232 (13)	0.047 (4)*	
H7	0.6898 (15)	0.059 (2)	0.7046 (14)	0.045 (4)*	
H8	0.5576 (11)	0.0536 (14)	0.9520 (10)	0.018 (3)*	
H9	0.6997 (13)	0.0194 (15)	0.9712 (11)	0.023 (3)*	
H10	0.4350 (13)	−0.1019 (18)	0.8023 (12)	0.032 (4)*	
H11	0.4723 (12)	−0.2806 (19)	0.7818 (13)	0.035 (4)*	
H12	0.4316 (13)	−0.2327 (17)	0.8900 (12)	0.033 (4)*	
H13	0.5616 (13)	−0.4628 (16)	0.9100 (11)	0.028 (4)*	
H14	0.7042 (13)	−0.6532 (18)	0.9862 (12)	0.032 (4)*	
H15	0.9091 (13)	−0.5706 (18)	1.0696 (11)	0.033 (4)*	
H16	0.9557 (12)	−0.3009 (15)	1.0693 (11)	0.021 (3)*	

### Atomic displacement parameters (Å^2^)

**Table:**

	*U*^11^	*U*^22^	*U*^33^	*U*^12^	*U*^13^	*U*^23^
N1	0.0190 (5)	0.0203 (5)	0.0189 (5)	0.0021 (4)	0.0054 (4)	0.0009 (4)
N2	0.0208 (5)	0.0187 (5)	0.0239 (5)	0.0052 (4)	0.0102 (4)	0.0059 (4)
N3	0.0177 (5)	0.0145 (5)	0.0211 (5)	−0.0003 (4)	0.0003 (4)	0.0002 (4)
N4	0.0185 (5)	0.0185 (5)	0.0166 (5)	0.0017 (4)	0.0035 (4)	0.0008 (4)
C1	0.0178 (6)	0.0235 (6)	0.0218 (6)	0.0025 (5)	0.0051 (5)	−0.0018 (5)
C2	0.0200 (6)	0.0186 (6)	0.0218 (6)	0.0038 (5)	−0.0002 (5)	−0.0010 (5)
C3	0.0260 (6)	0.0146 (6)	0.0170 (6)	−0.0035 (5)	0.0021 (5)	0.0005 (4)
C4	0.0186 (6)	0.0159 (6)	0.0177 (6)	−0.0029 (4)	0.0055 (5)	−0.0019 (4)
C5	0.0178 (6)	0.0133 (6)	0.0162 (5)	−0.0015 (4)	0.0037 (4)	−0.0029 (4)
C6	0.0236 (7)	0.0254 (7)	0.0363 (8)	0.0077 (6)	0.0145 (6)	0.0075 (6)
C7	0.0197 (6)	0.0149 (6)	0.0159 (6)	0.0004 (5)	0.0030 (5)	0.0000 (4)
C8	0.0196 (6)	0.0204 (7)	0.0274 (7)	−0.0006 (5)	−0.0007 (5)	−0.0002 (5)
C9	0.0208 (6)	0.0172 (6)	0.0120 (5)	0.0008 (5)	0.0063 (4)	0.0005 (4)
C10	0.0254 (7)	0.0185 (6)	0.0178 (6)	−0.0024 (5)	0.0067 (5)	−0.0015 (4)
C11	0.0400 (8)	0.0151 (6)	0.0208 (6)	0.0022 (5)	0.0111 (6)	0.0001 (5)
C12	0.0342 (7)	0.0219 (6)	0.0202 (6)	0.0119 (5)	0.0072 (5)	0.0039 (5)
C13	0.0224 (6)	0.0258 (7)	0.0173 (6)	0.0063 (5)	0.0044 (5)	0.0011 (5)

### Geometric parameters (Å, °)

**Table:**

N1—C1	1.3382 (15)	C4—H4	0.938 (15)
N1—C5	1.3431 (15)	C6—H5	0.983 (17)
N2—C5	1.3769 (15)	C6—H6	0.951 (18)
N2—C7	1.4485 (14)	C6—H7	0.998 (17)
N2—C6	1.4508 (15)	C7—H8	1.010 (13)
N3—C9	1.3773 (15)	C7—H9	0.968 (14)
N3—C8	1.4556 (15)	C8—H10	0.957 (16)
N3—C7	1.4563 (15)	C8—H11	0.996 (16)
N4—C13	1.3406 (15)	C8—H12	1.012 (16)
N4—C9	1.3463 (15)	C9—C10	1.4123 (16)
C1—C2	1.3823 (17)	C10—C11	1.3756 (17)
C1—H1	0.992 (15)	C10—H13	0.954 (14)
C2—C3	1.3907 (17)	C11—C12	1.3858 (19)
C2—H2	0.972 (14)	C11—H14	0.964 (15)
C3—C4	1.3753 (17)	C12—C13	1.3775 (18)
C3—H3	0.957 (15)	C12—H15	0.969 (15)
C4—C5	1.4121 (16)	C13—H16	1.001 (14)
			
C1—N1—C5	117.83 (10)	H6—C6—H7	108.0 (13)
C5—N2—C7	122.07 (9)	N2—C7—N3	112.76 (9)
C5—N2—C6	120.81 (10)	N2—C7—H8	107.5 (7)
C7—N2—C6	117.12 (9)	N3—C7—H8	108.9 (7)
C9—N3—C8	119.99 (10)	N2—C7—H9	109.8 (8)
C9—N3—C7	121.18 (9)	N3—C7—H9	107.0 (8)
C8—N3—C7	116.06 (10)	H8—C7—H9	111.0 (10)
C13—N4—C9	117.83 (10)	N3—C8—H10	107.8 (9)
N1—C1—C2	124.54 (11)	N3—C8—H11	109.9 (8)
N1—C1—H1	115.4 (8)	H10—C8—H11	110.5 (12)
C2—C1—H1	120.0 (8)	N3—C8—H12	111.2 (8)
C1—C2—C3	117.13 (11)	H10—C8—H12	107.2 (12)
C1—C2—H2	118.3 (8)	H11—C8—H12	110.2 (13)
C3—C2—H2	124.6 (8)	N4—C9—N3	117.13 (10)
C4—C3—C2	120.11 (11)	N4—C9—C10	121.76 (10)
C4—C3—H3	119.2 (9)	N3—C9—C10	121.10 (10)
C2—C3—H3	120.7 (9)	C11—C10—C9	118.39 (11)
C3—C4—C5	118.62 (11)	C11—C10—H13	119.9 (9)
C3—C4—H4	121.2 (9)	C9—C10—H13	121.6 (8)
C5—C4—H4	120.1 (9)	C10—C11—C12	120.22 (12)
N1—C5—N2	117.75 (10)	C10—C11—H14	118.5 (9)
N1—C5—C4	121.76 (10)	C12—C11—H14	121.3 (9)
N2—C5—C4	120.49 (10)	C13—C12—C11	117.55 (11)
N2—C6—H5	111.2 (10)	C13—C12—H15	118.8 (9)
N2—C6—H6	106.0 (10)	C11—C12—H15	123.7 (9)
H5—C6—H6	108.4 (14)	N4—C13—C12	124.25 (12)
N2—C6—H7	111.2 (10)	N4—C13—H16	116.8 (7)
H5—C6—H7	111.7 (14)	C12—C13—H16	118.9 (7)

### Hydrogen-bond geometry (Å, °)

**Table:**

*Cg*1 and *Cg*2 are the centroids of the N1/C1–C5 and N4/C9–C15 rings, respectively.

**Table:**

*D*—H···*A*	*D*—H	H···*A*	*D*···*A*	*D*—H···*A*
C1—H1···Cg2^i^	0.99 (2)	2.64 (2)	3.484 (1)	143 (1)
C6—H7···Cg2^ii^	1.00 (2)	2.75 (2)	3.545 (2)	137 (1)
C10—H13···Cg1^iii^	0.95 (2)	2.90 (1)	3.637 (1)	135 (1)

Symmetry codes: (i) −*x*+1, −*y*, −*z*+2; (ii) −*x*+3/2, *y*+1/2, −*z*+3/2; (iii) *x*, *y*−1, *z*.
